# Implementing electronic decision-support tools to strengthen healthcare network data-driven decision-making

**DOI:** 10.1186/s13690-020-00413-2

**Published:** 2020-06-18

**Authors:** Diego Rios-Zertuche, Alvaro Gonzalez-Marmol, Francisco Millán-Velasco, Karla Schwarzbauer, Ignez Tristao

**Affiliations:** 1 Salud Mesoamerica Initiative, Inter-American Development Bank, 1300 New York Ave NW, SE0631, Washington, DC 20577 USA; 2 Instituto de Salud del Estado de Chiapas, Tuxtla Gutiérrez, Mexico; 3 Salud Mesoamerica Initiative, Inter-American Development Bank, Tegucigalpa, Honduras; 4 Inter-American Development Bank, Buenos Aires, Argentina

**Keywords:** Decision-support tools, Maternal health, Health information systems, Decision-making, Data-driven, Evidence-based, Child health, Low-income countries, Middle-income countries, Data visualization

## Abstract

**Background:**

Ministries of health in low- and middle-income countries often lack timely quality data for data-driven decision making in healthcare networks. We describe the design and implementation of decision-support electronic tools by the Ministry of Health of the State of Chiapas, in Mexico, as part of Salud Mesoamerica Initiative.

**Methods:**

Three electronic decision-support tools were designed through an iterative process focused on streamlined implementation: 1) to collect and report health facility data at health facilities; 2) to compile and analyze data at health district and central level; and, 3) to support stratified sampling of health facilities. Data was collected for five composite indicators measuring availability of equipment, medicines, and supplies for maternal and child health. Quality Assurance Teams collected data, evaluated results and supported quality improvement. Data was also analyzed at the central level and health districts for decision-making.

**Results:**

Data from 300 health facilities in four health districts was collected and analyzed (November 2014—June 2015). The first wave revealed gaps on availability of equipment and supplies in more than half of health facilities. Electronic tools provided the ministry of health officers new ways to visualize data, identify patterns and make hypothesis on root-causes. Between the first and second measurement, the number of missing items decreased, and actions performed by quality improvement teams became more proactive. In the final measurement, 89.7—100% of all health facilities achieved all the required items for each indicator.

**Conclusions:**

Our experience could help guide others seeking to implement electronic decision-support tools in low- and middle-income countries. Electronic decision-support tools supported data-driven decision-making by identifying gaps on heatmaps and graphs at the health facility, subdistrict, district or state level. Through a rapid improvement process, the Ministry of Health met targets of externally verified indicators. Using available information technology resources facilitated prompt implementation and adoption of technology.

## Background

In spite of considerable agreement from the public health community that evidence-based decisions yield better health outcomes [1], ministries of health in low- and middle-income countries (LMICs) often lack timely quality data [2] or an organizational culture of data-driven decision making [2, 3]. Health information systems are highly fragmented [3–6], do not present timely information [2, 6–8], report inaccurate data [2, 6, 8], and lack the needed content and visualization formats required to support decisions [1, 6]. Further, ministries of health overlook the need for people with analytical profiles [5, 8, 9] and often lack leadership and coordination for information exchanges [3]. All of these challenges are present in Mesoamerica, a region encompassing the South of Mexico and Central America [10].

Technology can help address some of the outstanding issues of health information systems by facilitating and automating different aspects of data collection, analysis and use of data [2, 11]. Electronic decision-support tools have become increasingly popular. Decision-support tools “synthesize and display data to inform priority decisions [4].” These tools aim to improve decisions around diverse areas in health care, including planning, logistics and operations, healthcare provision, between others. Examples include electronic scorecards, dashboards, and electronic medical records with automated skip-patterns. Although several publications highlight the need for evidence-based decision making, there are only few examples of interventions to strengthen data-driven decision making [4, 9, 12]. While technology is important, its success relies on the effective implementation of decision-making mechanisms in which stakeholders have clear response times, roles, and responsibilities, and data is linked to actionable information. Most publications on decision-support tools in LMICs describe improvements in strategic planning or clinical decision-making, examples decision-support tools to improve decision-making in healthcare networks are limited [12].

In this paper, we describe the design and implementation of electronic decision-support tools by the Ministry of Health (MOH) of the State of Chiapas in the south of Mexico. The tools and technical assistance supported data collection, analysis and decision-making efforts throughout the healthcare network. This was motivated by the need to achieve targets of performance indicators in the context of a results-based aid program, the *Salud Mesoamerica Initiative* (SMI). The aim of this paper is to describe how the tools were developed and used, and what was the outcome of their implementation. The results of implementation illustrate the effects of data-driven decision-making.

## Methods

### Healthcare network in Chiapas

**Fig. 1:**
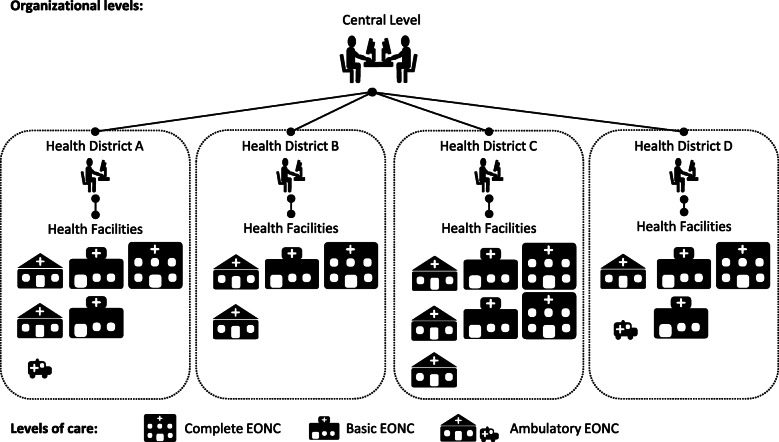
Ministry of Health Organizational Levels and Levels of Care. The Ministry of Health is organized in three organizational levels: 1) central level; 2) health districts; and, 2) health facilities. Health facilities can be categorized into four levels of Essential Obstetric and Neonatal Care (EONC): Ambulatory without a doctor, small facilities staffed with a nurse or auxiliary nurse that mainly provide basic antenatal and child car; ambulatory with a doctor, facilities staffed with a doctor and a nurse offering outpatient care; basic, facilities able to attend normal deliveries and provide initial emergency care; and complete, hospitals attending c-sections and resolving most obstetric and neonatal complications. Most health facilities report back to their corresponding district offices

Public health services in Mexico are decentralized to state governments. The role of the Federal Ministry of Health is mostly normative, establishing healthcare and guidelines, and partly financial. The MOH in Chiapas is responsible for health service provision. It’s healthcare network has three organizational levels: first, central level, in charge of planning, procurement, alignment with federal policies, and technical oversight; second, health districts that manage a geographic area encompassing between 40 to 140 health facilities of multiple levels of care; and, third, health facilities, including hospitals, clinics and mobile units (see Fig. 1). A wide range of services are offered, from community health promotion and prevention to highly complex medical care.

About 40% of the almost 5 million people living in Chiapas are served by the MOH, which makes it the largest healthcare provider [13]. Most of the population served is rural and poor. Chiapas is among the states with the highest poverty rates, lowest life expectancy and lowest per capita health expenditure [14]. It is also within the five states with highest maternal mortality [15]. The MOH operates over 1000 health facilities distributed along the state’s 28 thousand square miles.

### Health information Systems in Chiapas

Data routinely collected by the MOH in Chiapas is segmented, untimely, insufficient and underutilized. All health facilities report at least monthly (including the hardest-to-reach); however, manual record-keeping and labor-intensive compilation processes imply poor quality data and long compilation processes (2–3 months). To comply with federal and central level information requirements, data is siloed into multiple information systems: vital statistics, vaccinations, service production, infrastructure, and disease surveillance. Information on availability of supplies and quality of care is not routinely collected. Advanced data analysis is limited to epidemiology units, so data is not regularly used for benchmarking and performance improvement.

In terms of decisions for equipment, medicines and supplies, the central level makes procurement decisions considering yearly compilations of production data and historical trends. Commodities are distributed by district on mainly by-needs basis. Health facilities are mostly recipients and have a minimal role in the decision-making process, which is confined to reporting stock-outs.

### Salud Mesoamerica Initiative in Chiapas

SMI is a results-based aid program supporting countries as they strive to improve maternal and child health in the poorest areas. In Chiapas, the SMI program encompassed two phases lasting 24 months in four health districts, with a total of 301 health facilities. SMI and the MOH in Chiapas agreed to a set of indicators and targets for each phase. If the MOH met 80% of the agreed upon targets at the end of each phase, it could receive a performance award —US$1.9 million for the first phase. Indicators were measured at health facilities and followed an “all or nothing” rule, meaning that if any item from the composite indicator was missing for a given facility, the indicator received a value of zero for that facility. All indicators were externally and independently verified by the Institute for Health Metrics and Evaluation (IHME) from the University of Washington [16].

As the end of the first phase approached on 2014, the MOH had to make sure all health facilities were properly equipped and supplied with the basic maternal and child health inputs (see Additional file 1). A needs assessment conducted a year earlier to identify gaps and procure missing input. Yet, the MOH did not have the tools or mechanisms needed to know if anything was still missing or if inputs had been adequately distributed. Further, no communication mechanisms were established for health facilities to notify about missing inputs. The results of IHME’s external measurement showed that, although the MOH achieved significant improvements in availability of supplies and equipment between the baseline and the follow-up surveys, Chiapas was unable to meet targets and obtain the award [17].

The SMI Donors Committee established a Performance Improvement period, with an additional external measurement, for Chiapas to continue to the second phase. Considering the shorter timeframe, donors agreed that only indicators that were not met had to be measured. Over the following 9-months, the MOH implemented a rapid-improvement initiative, assisted by electronic decision-support tools, to effectively identify and close any remaining gaps [17].

Three electronic decision-support tools were designed through an iterative process focused on streamlined implementation. First, a Data Collection Tool (DCT) to gather and report data at the locally; second, a Data Analysis Tool (DAT) to compile and analyze data at health district and central levels; and, third, a Sampling Tool (ST) to support stratified sampling of health facilities. The design and implementation of the decision-support tools took advantage of a newly designed Quality Assurance (QA) Strategy. Although an ad-hoc design was necessary to meet the needs of the rapid-improvement initiative, the design had a broader focus of piloting the QA Strategy.

### New roles and responsibilities

During the first phase of the SMI program, SMI provided technical assistance designing a QA Strategy. The strategy shifted decision-making power towards health providers and health districts and encouraged a proactive approach to problems. Each organizational level was responsible for solving issues at their reach and following through on those raised to upper organizational levels. The strategy included transforming supervision teams into QA teams, with the new roles of measuring, evaluating and supporting quality of care at health facilities. Teams reported to health districts and, on average, oversaw a subdistrict health network with 20–30 health facilities. They were responsible for visiting health facilities and reporting findings at regular intervals. Teams met weekly with district managers to review the status of each issue and to raise to upper management those that could not be directly resolved. The central level and districts met periodically to resolve pending issues. Lastly, the strategy established common targets for the MOH, which corresponded to specific targets at health facilities, health district and central level. The strategy was endorsed by the Minister of Health and health district managers.

### Decision-support tools design and features

All the tools were designed in-house by one of the authors DRZ and programmed in MS Excel. MS Excel was widely available throughout the MOH and almost everyone had experience using it. QA teams were equipped with laptops and, since they were stationed in health districts (located in cities), they had Internet access and could send data daily.

#### Data collection tool

The DCT was designed to collect and report data per the criteria established by SMI indicators. The tool included the following features:

- A data collection checklist including all required equipment and supplies, in which the QA team recorded the date, if the item was available, not available or available but not functional, and described the finding and action taken. The checklist was color coded to track data collection progress and prevent submission of incomplete data. Data for five supplies, medicines and equipment indicators: antenatal care, delivery care, emergency care, child care, and family planning methods (for a detailed list of inputs for each indicators see Additional file 1). Different requirements were included depending on four levels of Essential Obstetric and Neonatal Care (EONC): Ambulatory without a doctor, small facilities staffed with a nurse or auxiliary nurse that mainly provide basic antenatal and child care; ambulatory with a doctor, facilities staffed with a doctor and a nurse offering outpatient care; basic, facilities able to attend normal deliveries and provide initial emergency care; and complete, hospitals attending c-sections and resolving most obstetric and neonatal complications [18].
- A report by health facility displaying a heat map of measurement results for each criterion by data collection date. Results could be displayed either for all indicators together or for each indicator individually. This display allowed the QA team to compare previous results with current results and discuss findings with health staff.
- A logbook showing the history of annotations for findings and actions taken at the health facility. If an input was consistently lacking, the QA team could review what actions had been taken.
- A subdistrict healthcare network report displaying a heat map with the latest measurement results of each facility within the specified time-period. Results could be displayed for indicators independently or together and filtered by level of care. This gave QA teams an overview of all health facilities under their responsibility and enabled producing different heat maps for different time periods.
- Function to export databases to facilitate sharing with the district level.
- Function to delete database, with an alert confirmation message to prevent accidental deletion.

The DCT was fully automated using macros and password protected to avoid format changes. The list of health facilities was preloaded with individual codes for each facility, so facilities could be clearly identified in the final database. At regular intervals, resulting data was sent by email to the district and the central level.

#### Data analysis tool

The DAT was designed to facilitate the analysis of aggregated data collected by all teams. The DAT incorporated different features, including:

- Instructions describing steps needed to update the database using individual files provided by QA teams (due to time constraints, this process was not automated).
- Summary report displaying data collection progress and aggregate indicator results. For example, line graphs, bar graphs, and tables showing the number of health facilities surveyed by date, the number of health facilities surveyed by health district, indicator results by level of care, among others.
- Detailed reports displaying results for criteria required by each indicator. For instance, reports included bar charts showing the percentage of health facilities with availability of each equipment or medical supply, and they showed how gaps affected the final indicator result.
- Findings and Actions report displaying the report recorded for all unmet criteria, which could be filtered by date, health facility level, health district or criteria. This helped identify systematic issues detected by multiple teams.

Given that the DAT was only meant to be used by specific people in four health districts and at the central level, the file was not password protected. All the tables and graphs were automated using Excel PivotTables tools, and the Findings and Actions report was automated using macros. The tables and graphs generated by the DAT could be easily compiled in a slide show to discuss results within districts or at the central level.

#### Sampling tool

The ST was designed to select a stratified random sample of health facilities. Health facilities were stratified according to each EONC level, which helped visualize challenges specific to each level of care and prioritize life-saving commodities. The user could input a sample size (equal or larger than 30), and the tool would automatically provide a stratified sample of health facilities.

### Training and pilot

After designing the DCT, training sessions and pilot tests with QA teams were performed. Training sessions were performed separately in each of the four districts. The first training was led by SMI and subsequent sessions were led by the MOH. Training took place through a 1-day session aimed at standardizing measurements followed by a 2-day pilot in the field. Training topics included an overview of the DCT, defining measurement criteria, and data collection from a fictional health facility. Given that the QA teams were proficient performing basic word and spreadsheet processing tasks (enter and edit data, change formatting, use menus, print), training to use the tool was minimal. During field visits, the QA teams were briefed to discuss findings with health facility staff and to act upon findings. In the pilot, software problems were also identified and corrected, and feedback from the users was obtained to improve the tool’s usability.

The DAT was designed during the initial implementation of the DCT. Training took place through a long-distance video-conference presentation, which mainly focused on compiling the data and creating visualizations. Once data became available, technical support was provided both on-site and long distance. No training was provided for the ST.

### Technical support

The implementation of all electronic tools was supported by two quality improvement consultants providing on-site technical assistance and long-distance technical support from SMI through online audio and video conferences. The consultants also accompanied and coached QA teams on field visits. Debugging was an important part of technical support. When issues were detected, a new version was made available ensuring compatibility with previously collected data.

### Rollout

Three waves of data collection and analysis were performed by the MOH. In the first two waves, the MOH performed a census of all health facilities. For each wave of data collection, a data collection plan with set deadlines was agreed between the central level and district leaders for each QA team to collect data from their subdistricts. In the first wave, the MOH collected data from 298 health facilities (November 2014—January 2015) and in the second wave of all 301 (February 2015—April 2015). In the third wave, a sample of 29 health facilities selected by the ST was collected (May 2015). The external survey to measure Performance Improvement results was collected by IHME a month after (June 2015).

## Results

### Decision-support tools findings

**Fig. 2:**
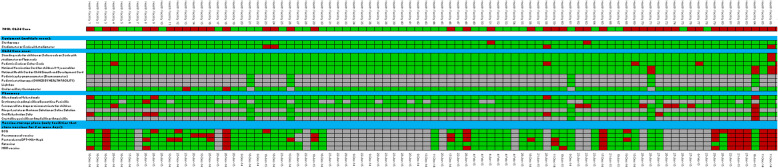
Heatmap of Inputs and Equipment for Child Care Observed in the First Wave of Data Collection, November 2014–January 2015. The image shows measurement results for equipment, medicines and supplies for child care in 298 health facilities. Each row represents an input (for example a stethoscope) and each column a health facility. Blue lines group categories of inputs: medicines, equipment and vaccines. Colors in cells represent if the required item was available: green shows that it was observed, red that it was not observed, and grey that it did not apply for that facility. The top row shows the overall indicator result for each facility

**Fig. 3:**
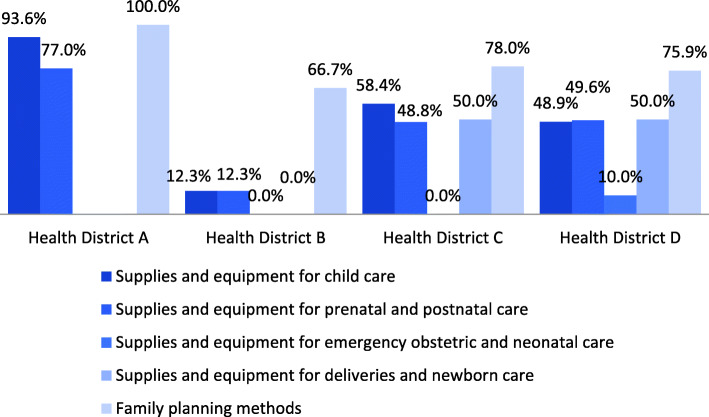
Indicator Results by Health District, First Wave, November 2014—January 2015. Results for inputs and equipment indicators by health district. Only three indicators were measured in District A

The first measurement wave revealed gaps on availability of equipment and supplies in an important number of health facilities in the network. In most facilities, only one or two items were missing, which was driving down the final values of indicators given the “all or nothing” criteria. Figure 2 shows a sample heat map created by the DCT displaying the results of the first wave. Although indicator results varied widely across districts (see Fig. 3), common issues were identified. For example, electronic scales available in most health facilities required batteries or a power cord to operate, which left scales inoperative. Other examples included gynecological examination tables without leg rests; and vaccines only available for routine programming —without stock during the week for walk-ins. Several issues identified could be solved locally, such as contraceptives and drugs available at district warehouses, lamps requiring bulb replacements and equipment requiring basic maintenance.

Electronic tools provided central level and district leaders new ways of analyzing and visualizing data. Decisions were customized and prioritized considering issues affecting a large proportion of health facilities, gaps on life-saving commodities, stockouts between health districts or for specific levels of care. Decisions were made considering if local solutions were possible or a central level response was required. Central level and district leaders were able to identify patterns and make hypotheses on root causes. While indicators were used strategically to track overall progress and compare heath districts, they also had operational definitions allowing district leaders and QA teams to act upon findings. QA teams created a feedback mechanism to ensure implementation compliance and bring back issues reported by service providers. Central and district leaders not only made decisions to address gaps but could check if their decisions were leading to the desired outcomes.

**Fig. 4:**
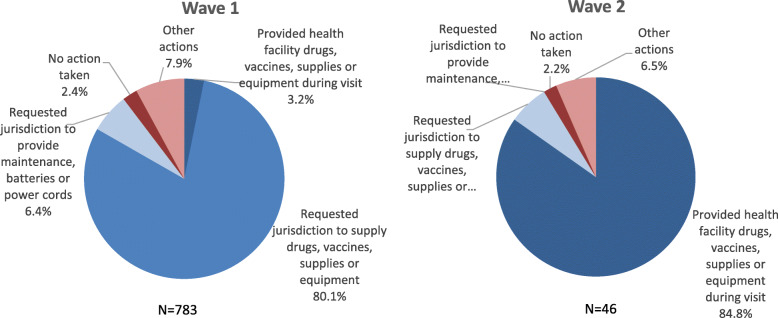
Summary of Actions Reported, First and Second Wave. Comparison of actions reported on the Data Collection Tool for the two initial waves of data collection: first wave, November 2014–January 2015; second wave, February 2015–April 2015. Recorded actions reported were grouped into categories

The analysis of findings and actions reported by QA teams in the first and second wave of data collection illustrates a shift in decision-making. The number of findings decreased, as it is expected with an improving situation, and the actions recorded became more proactive. On the first wave of data collection, only 3% of the actions implied addressing the finding directly (providing supplies or equipment), on the second wave the proportion increased to 85% of all actions. Figure 4 shows a summary of the findings and actions taken by QA teams during the first and second waves of data collection (recorded findings and actions were grouped into categories to facilitate the analysis).

**Fig. 5:**
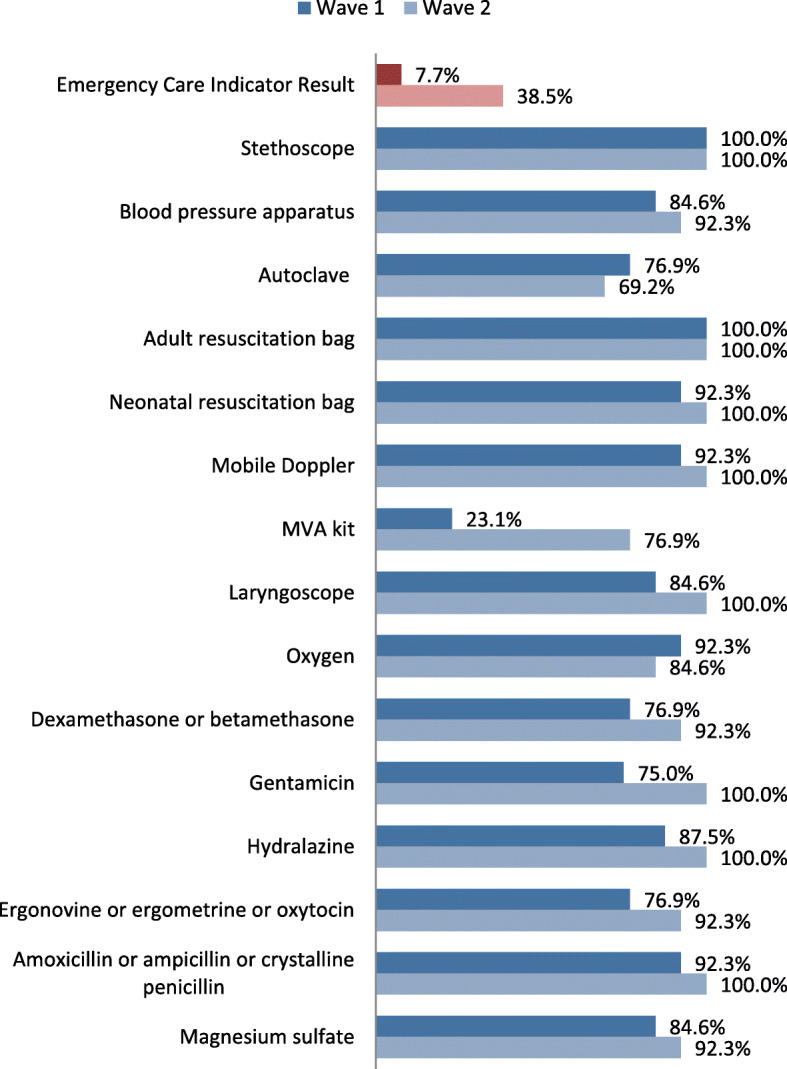
Indicator Progress Between First and Second Wave of Data Collection. Comparison of Supplies and Equipment for Emergency Obstetric and Neonatal Care indicator on the two initial waves of data collection: first wave, November 2014–January 2015; second wave, February 2015–April 2015. The bars show the percentage of health facilities in which supplies and equipment were observed. Availability of the complete set of items was needed for a facility to comply with indicator criteria

Availability of equipment and supplies improved progressively in health facilities. Although important progress was made between the first and second wave of data collection, the MOH believed it was not enough to meet targets. Figure 5 illustrates how inputs for emergencies improved between waves. After QA teams reported that health providers were not fully aware of the required inputs, the MOH decided to print banners for each health facility with the list of equipment and supplies needed and instructions on how to proceed if something was missing.

### MOH measurements and external measurements

**Fig. 6:**
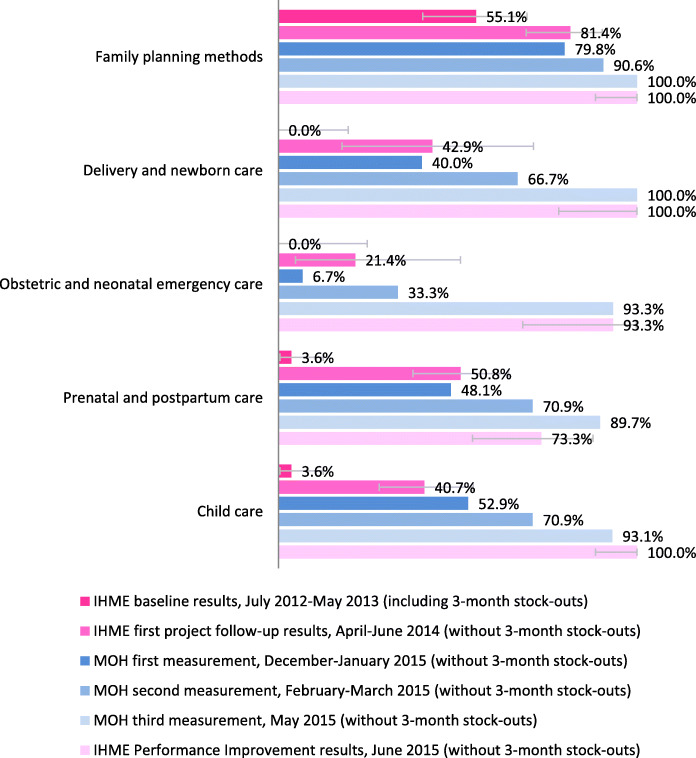
IHME Survey results and MOH measurement results. Comparison between IHME’s Health Facility Survey results and the three measurements by the Ministry of Health for indicators on availability of equipment and supplies. All surveys by IHME were collected from a sample of health facilities. The two first measurements by the MOH were a census of health facilities in SMI target areas, and the third measurement was collected on a sample of 30 facilities. Error bars show 95% Confidence Intervals

The Ministry of Health met the targets for all indicators in IHME’s Performance Improvement measurement. Results of the three waves collected by the MOH were consistent with each other and with IHME’s external measurements, showing progressive improvements between measurements. Figure 6 compares the three external measurements by IHME with the three waves collected by the MOH. In 3 out of 5 indicators, the difference in results between IHME’s first project follow-up survey and the first wave of data collection by the MOH was less than 5 percentage points. The results of all indicators in the first wave were also within the confidence intervals of IHME’s survey for all indicators. Even when the third wave came from a small sample, measurement results from IHME and the MOH were highly aligned; only one indicator was outside IHME’s survey confidence interval.

### Adoption of the decision-support tools

After the intense work performed during the Performance Improvement period, the MOH acknowledged the value of data to improve service delivery. The MOH decided to bring to scale the Quality Assurance Strategy to the whole State, including the creation of QA teams in all districts. As part of this endeavor, the MOH information technology (IT) team was appointed to create data collection and analysis tools based on the DCT and DAT developed by SMI. The MOH’s IT team created a cloud-based system incorporating many features, such as an intelligent web form for data collection, a log for findings and actions, automatic visualization of heat maps, among others. The MOH is currently developing an Android application to allow for offline data collection and analysis, which could be used by QA teams to collect data in health facilities without Internet access. These tools are being developed under the MOH’s own initiative and integrated into their dashboard and IT infrastructure. Preliminary versions of their open-source software are freely available online [19].

## Discussion

To our knowledge, few examples of decision-support tools have been implemented at scale to strengthen data-driven decision-making throughout a health network in LMICs [4, 12]. The use of decision-support tools, together with decision-making mechanisms, with clear response times, roles and responsibilities for each organizational level (central, district and health facilities), empowered the MOH in Chiapas to improve rapidly and achieve indicator targets in a short timeframe. Features introduced by the electronic decision-support tools were appropriated by the MOH and adopted to continually monitor service delivery outside of SMI, pointing to a sustained effect of their implementation on the MOH’s decision-making.

The fast and progressive improvements in indicators draw multiple lessons from implementation. First, decision-support tools were not implemented in isolation and addressed the complete data cycle (seek, collect, analyze, act, evaluate). A technological solution alone might not have produced the same results. The deployment of the tools in-line with the MOH strategic priorities was essential. The QA Strategy had already achieved political support among the MOH. QA teams had been certified on quality assurance, and quality of care had been widely discussed by district and central-level officials. Hence, all stakeholders understood their roles and responsibilities. The electronic decision-support tools facilitated the operation and integration of the strategy throughout the health network. In addition, the implementation process was well-rounded, including systematic processes to construct indicators, collect data, analyze results, and use the information for decision-making in a short timeframe, which enabled a high level of alignment across organizational levels. In just 9-months, the MOH collected 3 waves of data and improved indicators in 300 health facilities.

From a technological perspective, minimal investment and little training was needed given that the software (MS Excel) and hardware (laptops) used were already available across the MOH. Self-confidence is an important predictor for technology use [20], so using existing software also decreased adoption barriers. Furthermore, we avoided timely procurement processes and seized the opportunity created by SMI. Lastly, we were able to test the concept without a long-term commitment from the MOH. The Ministry’s IT team reviewed the tools’ front and back-end and scrutinized their features and functionality. Their close involvement avoided creating a parallel system relying on data recorded by health providers (as most information systems operate).

We observed that the quality of the data collected by the MOH was consistent with external measurements. Data quality and timeliness were vital for effective decision-making and supported the appropriation of the tools by the MOH after the Performance Improvement period. The small differences between the external survey and MOH measurements increased confidence of MOH senior officials on the ministry’s capacity to collect quality data.

Although the MOH has adopted and continues to develop open-source electronic decision-support tools, it is important to acknowledge structural threats for long-term sustainability. Only a few officials within the MOH have the strong analytical and methodological skills and managerial perspective to guide the electronic tools’ future expansion. High staff turnover, rotation and government changes are an additional threat. As has been suggested in studies about health information systems [1, 2, 5, 9], there is a pressing need to build capacity for health information. On the other hand, sustaining data quality on the long term may prove challenging. The impetus for improvement and rapid decision-making that we observed during the Performance Improvement period may wear off. If QA teams and health providers perceive that nobody is acting upon findings, data quality could be affected. Likewise, perverse incentives could be generated if data is used to penalize health providers.

As we have shown, by implementing the decision-support tools, the MOH met the targets for the performance indicators established by SMI. The need for rapid improvement created by SMI was instrumental for the successful implementation of the decision-support tools. The political visibility of SMI and the MOH’s motivation to achieve indicator targets accelerated decision-making and decreased barriers. Our experience supports the idea that performance-based initiatives may be catalytic to encourage data-driven decision-making [5]. We speculate whether the implementation of our electronic decision-support tools could be successful in other contexts.

## Conclusion

We hope that our experience will help others seeking to implement electronic decision-support tools in in LMICs. Electronic decision-support tools planted a seed in Chiapas for data-driven decision-making. The MOH was able to successfully undertake a rapid improvement process and met targets of externally verified indicators. The use of available IT resources facilitated prompt implementation and the adoption and appropriation by the MOH. Success was based on strong alignment between the electronic tools and MOH strategies and on establishing processes for the complete data cycle.

## Supplementary information

**Additional file 1.** Equipment, Medicines and Supplies Requirements by Indicator.

**Additional file 2.** Data Collection Tool and Database.

## Data Availability

All data generated or analyzed during this study are included in this published article and its supplementary information files (see Additional file 2).

## Abbreviations

DAT: Data Analysis Tool
DCT: Data Collection tool
EONC: Essential Obstetric and Neonatal Care
IHME: Institute for Health Metrics and Evaluation, University of Washington
LMICs: Low and Middle Income Countries
MOH: Ministry of Health
MS Excel: Microsoft Excel
QA: Quality Assurance
SMI: Salud Mesoamerica Initiative
ST: Sampling Tool
