# Supplementary material for: Implementing electronic decision-support tools to strengthen healthcare network data-driven decision-making
Source: Arch Public Health. 2020 Jun 18;78:33. doi: 10.1186/s13690-020-00413-2 (PMC7301503; doi:10.1186/s13690-020-00413-2)
Supplement: Supplementary file 1 — Additional file 1. Equipment, Medicines and Supplies Requirements by Indicator. [file 13690_2020_413_MOESM1_ESM.pdf]

Additional file 1: Implementing electronic decision-support tools to strengthen healthcare network data-driven decision-making

Equipment, Medicines and Supplies Requirements by Indicator

Contents

1. Supplies and equipment for antenatal and postpartum care 1

2. Supplies and equipment for delivery care 2

3. Supplies and equipment for emergency care 3

4. Supplies and equipment for child care 4

5. Family planning methods 6

1. Supplies and equipment for antenatal and postpartum care

Ambulatory EONC (without doctor)

- Iron and folic acid or multivitamin
- Standing scales
- Stadiometer or tallimeter
- Gynecological exam table
- CLAP obstetric tape
- Lamp gooseneck or hand lamp
- Sphygmomanometer
- Stethoscope
- Perinatal maternal medical history
- Perinatal maternal card

Ambulatory EONC (with doctor)

- Iron and folic acid or multivitamin
- Erythromycin or ampicillin or penicillin benzathine
- Tetanus vaccine
- Ayre palettes
- Microscope slides
- Nitrofurantoin
- Standing scales
- Stadiometer or tallimeter
- Gynecological exam table
- CLAP obstetric tape
- Lamp gooseneck or hand lamp
- Sphygmomanometer
- Stethoscope
- Set for IUD insertion
- Perinatal maternal medical history
- Perinatal maternal card

Basic EONC

- Iron and folic acid or multivitamin
- Cephalexin
- Tetanus vaccine
- Ayre palettes
- Microscope slides
- Nitrofurantoin
- Standing scales
- Stadiometer or tallimeter
- Gynecological exam table
- CLAP obstetric tape
- Lamp gooseneck or hand lamp
- Sphygmomanometer
- Stethoscope
- Set for IUD insertion
- Perinatal maternal medical history
- Perinatal maternal card

Complete EONC

- Iron and folic acid or multivitamin
- Cephalexin
- Tetanus vaccine
- Ayre palettes
- Microscope slides
- Nitrofurantoin
- Standing scales
- Stadiometer or tallimeter
- Gynecological exam table
- CLAP obstetric tape
- Lamp gooseneck or hand lamp
- Sphygmomanometer
- Stethoscope
- Set for IUD insertion
- Perinatal maternal medical history
- Perinatal maternal card

2. Supplies and equipment for delivery care

Basic EONC

- Hyoscine bromide
- Plastic clamp or umbilical tape
- Ergonovine maleate or ergometrine or oxytocin
- Drops of chloramphenicol ophthalmic or 1% silver nitrate or oxytetracycline ophthalmic
- Ringer or Hartman solution or saline solution

- Lidocaine or epinephrine
- Insulin syringe
- Vitamin K
- Intravenous catheter sterile No 18
- Metallic clamp or umbilical tape
- Equipment for serum
- Nasogastric tube
- Sterile fields or sheltering or baby

Complete EONC

- Hyoscine bromide
- Plastic clamp or umbilical tape
- Ergonovine maleate or ergometrine or oxytocin
- Drops of chloramphenicol ophthalmic or 1% silver nitrate or oxytetracycline ophthalmic
- Ringer or Hartman solution or saline solution
- Lidocaine or epinephrine
- Insulin syringe
- Vitamin K
- Intravenous catheter sterile No 18
- Metallic clamp or umbilical tape
- Equipment for serum
- Nasogastric tube
- Sterile fields or sheltering or baby

3. Supplies and equipment for emergency care

Basic EONC

- Penicillin crystals or IV ampicillin or amoxicillin
- Dexamethasone or betamethasone
- Gentamicin
- Hydralazine
- Magnesium sulfate
- Oxytocin or ergometrine
- Autoclave or dry heat sterilizer
- Sphygmomanometer
- Laryngoscope
- MVA kit
- Oxygen tank or oxygen connection
- Portable doppler or Pinard stethoscope
- Adult reanimation bag
- Neonatal resuscitation bag
- Stethoscope

Basic EONC

- Amikacin or amikacin sulfate

- Penicillin crystals or IV ampicillin or amoxicillin
- Ceftriaxone
- Chloramphenicol or metronidazole
- Dexamethasone or betamethasone
- Diazepam or midazolam chloral hydrate
- Magnesium sulfate
- Nifedipine
- Oxytocin or ergometrine
- Sevoflurane
- Succinylcholine chloride
- Anesthesia equipment
- Autoclave or dry heat sterilizer
- Sphygmomanometer
- Kit for C-sections
- Laryngoscope
- MVA kit
- Neonatal or pediatric stethoscope
- Oxygen tank or oxygen connection
- Portable doppler or Pinard stethoscope
- Adult reanimation bag
- Neonatal resuscitation bag
- Stethoscope

4. Supplies and equipment for child care

Ambulatory EONC (without doctor)

- Packet or envelopes of oral rehydration salt
- Ferrous sulfate drops
- Albendazole or mebendazole
- Pediatric balance or scale
- Standing balance or scale for children
- Tallimeter or stadiometer
- Stethoscope
- Oral or axillary thermometer
- Growth card

Ambulatory EONC (with doctor)

- Packet or envelopes of oral rehydration salt
- Ferrous sulfate drops
- Albendazole or mebendazole
- Erythromycin or ampicillin or penicillin benzathine
- Pediatric balance or scale
- Standing balance or scale for children
- Tallimeter or stadiometer
- Stethoscope

- Oral or axillary thermometer
- Growth card
- Pentavalent vaccine
- MMR vaccine
- Rotavirus vaccine
- Pneumococcal conjugate vaccine
- BCG vaccine

Basic EONC

- Packet or envelopes of oral rehydration salt
- Ferrous sulfate drops
- Albendazole or mebendazole
- Crystalline penicillin or ampicillin or amoxicillin
- Ringer or Hartman lactate or saline solution
- Pediatric balance or scale or salter scale
- Standing balance or scale for children
- Stadiometer or tallimeter
- Pediatric tensiometer
- Pediatric stethoscope
- Growth card
- Pentavalent vaccine
- MMR vaccine
- Rotavirus vaccine
- Pneumococcal conjugate vaccine
- BCG vaccine

Complete EONC

- Packet or envelopes of oral rehydration salt
- Ferrous sulfate drops
- Albendazole or mebendazole
- Crystalline penicillin or ampicillin or amoxicillin
- Ringer or Hartman lactate or saline solution
- Pediatric balance or scale or salter scale
- Standing balance or scale for children
- Stadiometer or tallimeter
- Pediatric tensiometer
- Pediatric stethoscope
- Growth card
- Pentavalent vaccine
- MMR vaccine
- Rotavirus vaccine
- Pneumococcal conjugate vaccine
- BCG vaccine

5. Family planning methods

Ambulatory EONC

- Male condom
- Any oral contraceptive
- Any injectable contraceptive

Basic EONC

- Male condom
- Any oral contraceptive
- Any injectable contraceptive
- IUD
- IUD insertion kit

Complete EONC

- Male condom
- Any oral contraceptive
- Any injectable contraceptive
- IUD
- IUD insertion kit
- Doctor trained to perform tubal ligation & vasectomy
